# A brief overview about the adipokine: Isthmin-1

**DOI:** 10.3389/fcvm.2022.939757

**Published:** 2022-07-26

**Authors:** Min Hu, Xin Zhang, Can Hu, Teng Teng, Qi-Zhu Tang

**Affiliations:** ^1^Department of Cardiology, Renmin Hospital of Wuhan University, Wuhan, China; ^2^Hubei Key Laboratory of Metabolic and Chronic Diseases, Wuhan, China

**Keywords:** adipokine, isthmin-1, distribution, structure, receptor, biological function

## Abstract

Isthmin-1 is a secreted protein with multiple capability; however, it truly attracts our attention since the definition as an adipokine in 2021, which exerts indispensable roles in various pathophysiological processes through the endocrine or autocrine manners. In this review, we summarize recent knowledge of isthmin-1, including its distribution, structure, receptor and potential function.

## Introduction

Adipokines are bioactive peptides secreted by adipose tissue, which mainly act locally on adipose tissue in the paracrine or autocrine manners or act on distant target organs (e.g., heart, lung, bone, pancreas, brain and liver, etc.) to regulate their growth, development, metabolism and tissue reconstruction through an endocrine way (1–3). What's more, adipokines participate in many pathophysiological processes, such as immune response, lipid metabolism, inflammation, vasoactivity and oxidative stress (4–6). Moreover, our previous studies revealed that C1q/tumor necrosis factor-related protein-3, an adipokine and a paralog of adiponectin could protect against sepsis-, doxorubicin- induced myocardial dysfunction but exacerbate cardiac hypertrophy in mice (7–9). Besides, our previous studies identified that fibronectin type III domain-containing 5 played a crucial role in alleviating oxidative stress and cardiomyocyte apoptosis in doxorubicin-induced cardiotoxicity and improving aging-related cardiac dysfunction (10, 11). On the other hand, our co-workers certified that meteorin-like protein, a newly identified myokine downregulated in DOX-treated murine hearts and cardiomyocytes, could attenuates doxorubicin-induced cardiotoxicity (12). Through unbiased secretion cloning screen, Isthmin-1(ISM1) was initially identified as a secreted protein according to its prominent expression at the isthmus of *Xenopus* embryos in 2002 (13). Significantly, ISM1 was defined as an adipokine in 2021 combining bioinformatic analyses with expression data on mature brown and white adipocytes, which played an important role in glucose uptake and lipid metabolism (14). ISM1 belongs to isthmin gene family that contains ISM1 and ISM2 (also known as Tail1). ISM1 located on chromosome 20, while the ISM2 located on chromosome 14q24.3 in human, both of the isthmin genes encode secreted proteins containing TSR1 and AMOP domains (15, 16). ISM1 is widely distributed in different body compartments and is involved in many pathophysiological processes, such as metabolism, immunization, tumorigenesis, cell growth, permeability of endothelial cells and physiogenesis. ISM2 is mainly expressed in the placenta and is associated with choriocarcinoma and preeclampsia (17). Given the wide distribution and extensive roles of ISM1, we present the current knowledge on the structure, distribution, receptor and function of ISM1 in this review.

## Location, gene and protein structure

**Figure 1:**
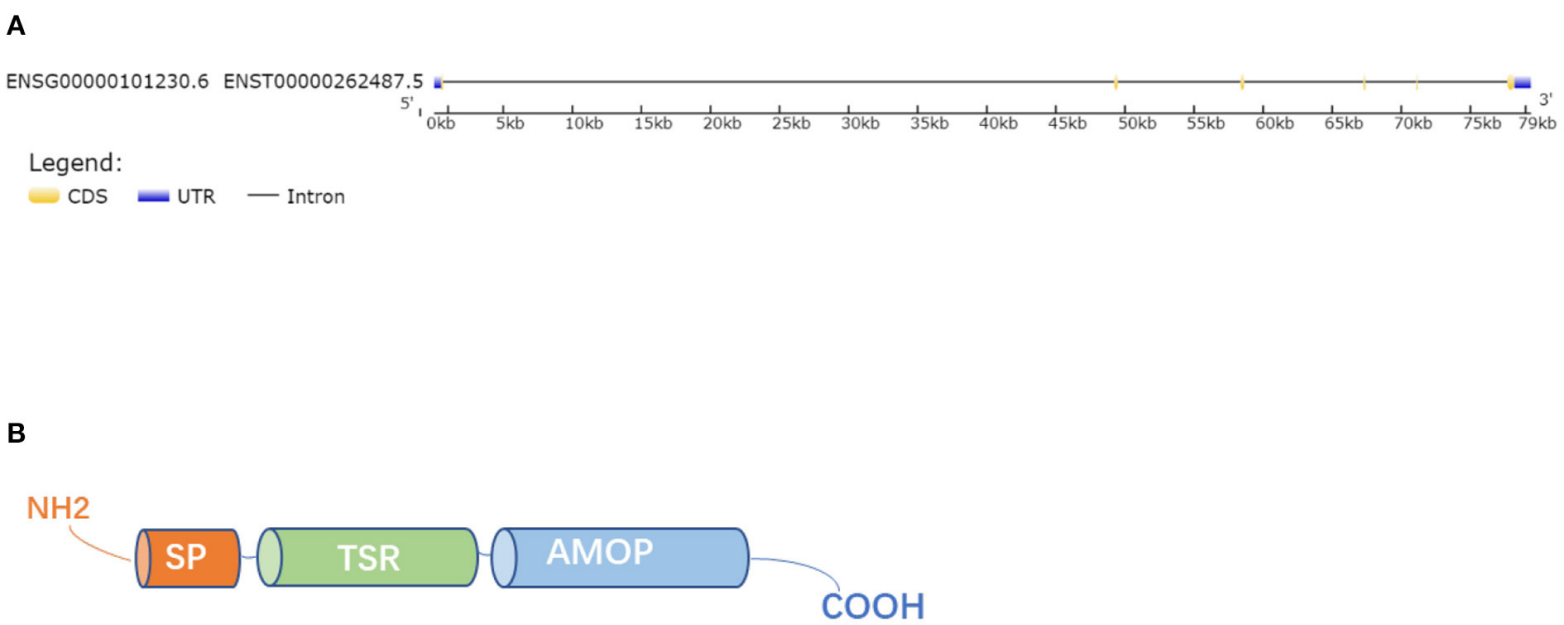
Gene and protein structure. **(A)** The gene structure of ISM1. **(B)** The whole ISM1 protein was composed of an amino (N)-terminal signal peptide (SP), a TSR domain and a conserved C-terminal AMOP domain.

Human ISM1 gene is located on chromosome 20 and encodes a ~60 kDa protein with 499 amino acids that contains 3 α-helices and 2 β-sheets (16). Mouse ISM1 gene is located on chromosome 2 and encodes a 461 amino acid protein with a predicted molecular weight of 52 kDa. In addition, Liliana et al. (18) identified that there was a high homology between ISM1 orthologs by Clustal Omega analysis, especially, mouse ISM1 shared 93 percent of identity with human. Protein of ISM1 was composed of an amino (N)-terminal signal peptide (SP), one copy of the thrombospondin type 1 repeat (TSR) in the central region and a conserved C-terminal adhesion-associated domain in MUC4 and other proteins region (AMOP) (16) (Figure 1). The TSR and AMOP domains of ISM1 are highly conserved among species. The TSR domain is highly conserved with 98% homology between mouse and human, 87–88% homology between mouse and zebrafish or mouse and Xenopus. The C-terminal AMOP domain is highly conserved with 99% homology between mouse and human, and mouse is 91% identical with *Xenopus* and 85% identical with zebrafish (19). TSR and AMOP have been found in many secreted proteins or the extracellular portions of transmembrane proteins (20, 21). The TSR is related to cell migration, communication and tissue remodeling while the AMOP is involved in cell adhesion and angiogenesis (22, 23). The TSR domain of ISM1 contains two consensus C-mannosylation sequences at Trp^223^ and Trp^226^, which not only affects the secretion of ISM1 but also affects its N-glycosylation (24). The AMOP domain of ISM1 may mediate angiogenesis by regulating the interaction between ISM1 and αvβ5 integrin, function as proapoptotic ligands of cell-surface glucose-regulatory protein 78KDa (GRP78) (18, 19, 25). Besides, AMOP can mediate the interaction between ISM1 and human umbilical vein endothelial cells (ECs) and NODAL signaling (26).

## Expression pattern

It is well-accepted that ISM1 is abundantly expressed in the early embryo and its expression displays dynamic alterations (13, 19). ISM1 transcripts could be observed in E6.75 embryos and the spatial distribution of ISM1 transcripts broaden as the embryo develops. What's more, the expression of ISM1 in the developing somite is limited to the dermomyotome, where stronger expression is observed in more posterior regions than anterior positions in mice (18). ISM1 may be important for proper development due to its specific expression pattern. After birth and in adulthood, ISM1 is widely distributed in all parts of body, especially in the brain and lungs, where strongly expression is detected in the epithelium of the bronchi and alveoli as well as in the cerebellum, cerebral cortex, and hippocampus of the brain. Besides, organs such as the eye, kidney, skeletal muscle, and heart also express ISM1 to varying degrees (18). On the other hand, ISM1 may be related to the NK, NKT, AND Th17 cell lineages in human and mice. ISM1 has been found to be produce by the immune barrier like skin, mucosal tissue and selected lymphocyte populations, specially, the expression of ISM1 in CD4^+^ T cells increases significantly when the cells are polarized to the Th17 lineage *in vitro* (27). Collectively, ISM1 is a regulator of lymphocyte effector functions and may be involved in innate and adaptive immune responses. Furthermore, robust expression of ISM1 has been identified in isolated mature adipocytes verified by RNA and protein analyses, and the cycling levels of ISM1 are physiologically regulated by nutritional and metabolic changes in mice and humans, suggesting an important role of ISM1 in various pathophysiological processes as an adipokine (14).

## Receptor

**Figure 2:**
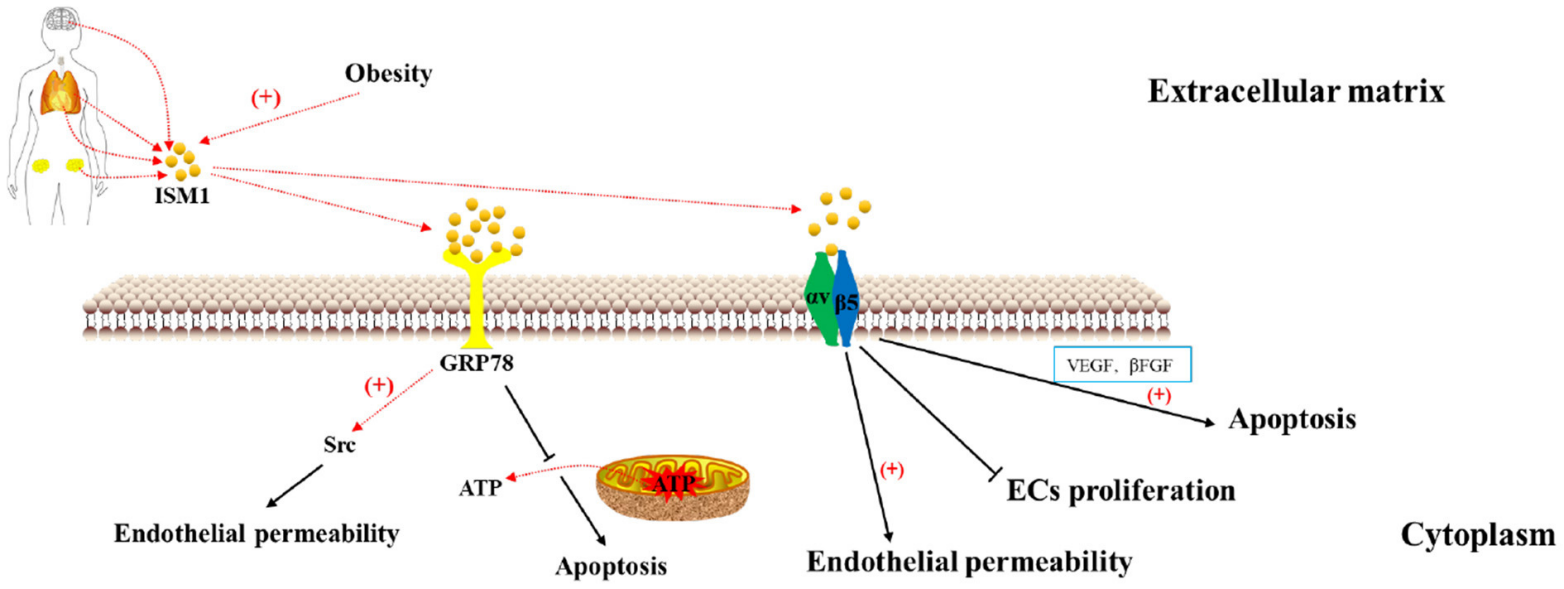
ISM1 binds to GRP78 and αvβ5 integrin to induce apoptosis and promote endothelial permeability.

Two potential receptors for ISM1 including the low-affinity αvβ5 integrin and the high-affinity GRP78 have been previously identified. Using ligand binding or functional assays, the affinity of agonists binding to the receptor can be estimated by the dissociation constant. The lower the dissociation constant is, the stronger the affinity is (28–30). Integrins are a class of transmembrane receptors mediating cell adhesion to matrix molecules and play important roles in angiogenesis and inflammation (31, 32). Using a solid-phase ELISA-based binding assay, Zhang et al. identified that ISM1 bound αvβ5 integrin with a Kd around 40 uM, within the range of integrin ECM ligand binding affinities. ISM1 binds to EC surface αvβ5 integrin through a novel “RKD” motif located in 315–317 amino acids of the C-terminal AMOP domain (33). GRP78 is traditionally regarded as an ER lumen chaperon protein for facilitating protein folding and mediating cellular stress response (34). Moreover, a portion of the GRP78 is present on the cell surface especially when overexpression of the ER form of GRP78 or under ER stress, which serve as receptors for proapoptotic ligands such as Kringle-5 (K5) and Par-4 (35–37). Chen et al. (38) found that GRP78 was a high-affinity receptor for ISM1 and the binding affinity between recombinant GRP78 (rGRP78) and recombinant ISM1 (rISM1) was high with a Kd around 8.58nM. Kao et al. (25) reported that novel cyclic peptides harboring the RKD motif in the ISM1 AMOP domain were proapoptotic ligands of cell-surface GRP78. Surface GRP78 is preferentially present in cancer cells, it is an attractive target for cancer therapy. In addition, the expression level of GRP78 on the surface of normal and benign tumor cells was low and resistant to ISM1-induced apoptosis (38). What's important, both of the receptors can mediate ISM1 internalization and accelerate apoptosis of activated EC cells and tumor cells (19, 38) (Figure 2).

## Biological function

**Figure 3:**
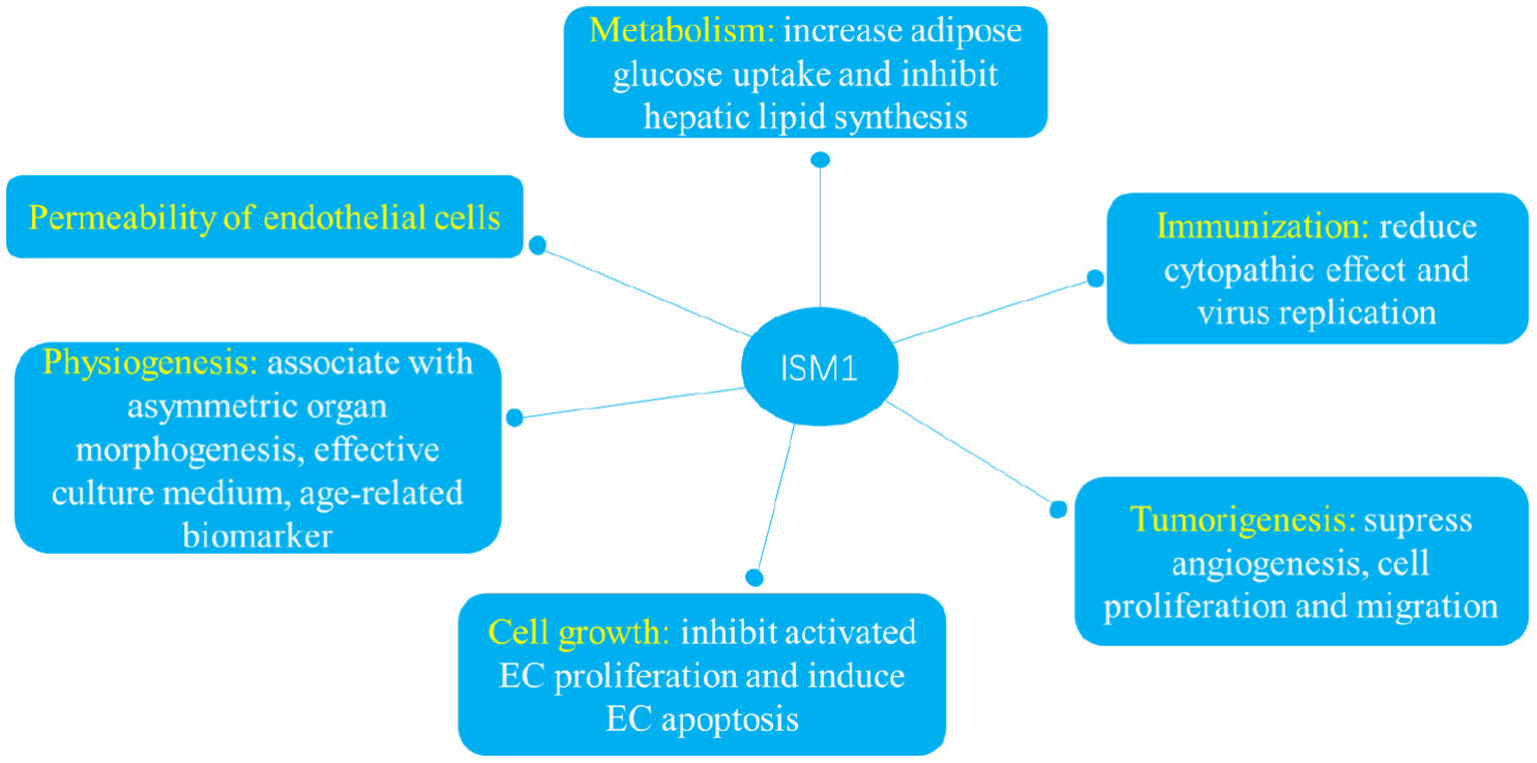
ISM1 plays indispensable roles in a variety of pathophysiological processes including immunization, tumorigenesis, cell growth, permeability of endothelial cells and physiogenesis.

Many indispensable roles have be ascribed to ISM1 since its first identification in 2002 (13). ISM1 is not only involved in metabolism as an adipokine but also plays indispensable roles in a variety of pathophysiological processes including immunization, tumorigenesis, cell growth, permeability of endothelial cells and physiogenesis (Figure 3).

### Metabolism

Metabolism consists of a series of reactions that mainly involved in cellular pathways to sustain life (39). Metabolic flexibility is important for responding or adapting to conditional changes in metabolic demand, otherwise metabolic diseases like diabetes and fatty liver will proliferate (40). It has recently been shown that ISM1 played an essential role in diabetes mellitus and fatty liver disease as an adipokine. By comparing glucose tolerance and liver fat synthesis in overexpressed or knockdown ISM1 mice, Jiang and co-workers verified that ISM1 had a dual role in increasing adipose glucose uptake while inhibiting hepatic lipid synthesis (14). Nevertheless, few of trials have evaluated the therapeutic potential of ISM1 as an adipokine in human disease, and this should be the main research goal for the near future.

### Immunization

Innate immunity, known as non-specific immunity, serves as the first line of defense of host against the invasion of pathogens and recent studies found that ISM1 might be a new target for controlling viral infection in antiviral immune response (41, 42). Ricardo et al. (27) found that ISM1 had a barrier function in mouse and human with significant expression in mucosal and skin. An emerging study proved that the expression of ISM1 in zebrafish challenged with GCRV was inducible, and rISM1 could reduce cytopathic effect and virus replication, where ISM1 was able to promote the expression of antiviral genes such as IFN and MX *via* TBK1–IRF3–IFN signaling pathway (16).

### Tumorigenesis

An increasing number of studies have shown that ISM1 participates in the progression of tumorigenesis, and also regulates the migration and invasion of tumor cells, especially in solid tumors such as melanoma, hepatocellular cancer, colorectal cancer (CRC) and breast cancer, all of which are known to be angiogenesis dependent (19, 43, 44). Xiang et al. (19) verified that ISM1 was a novel secreted angiogenesis inhibitor which inhibited EC capillary network formation on Matrigel through its C-terminal AMOP domain, and ISM1 overexpression significantly suppressed mouse B16 melanoma tumor growth through inhibiting angiogenesis but barely affected tumor cell proliferation. Additionally, Wang and co-workers identified that ISM1 could suppress cell proliferation and migration in hepatocellular cancer, which was regulated by hsa_circ_0091570/miR1307 (45). A similar article by Zheng et al. (44) found that miR-1307–3p inhibited activation of Wnt3a/β-catenin signaling through downregulating ISM1, thereby inhibited proliferation and promoted apoptosis of colon adenocarcinoma cells. Through investigating the expression of ISM1 in 18 pairs CRC tissues from GSE50760, and 473 CRC tissues vs. 41 normal tissues from The Cancer Genome Atlas (TCGA), Wu and co-workers found that ISM1 was upregulated in CRC tissues and was significantly associated with multiple cancer-related pathways and immune-related pathways such as EMT, hypoxia, and the Notch and KRAS signaling pathways (46). It's well known that tumor DNA methylation profiling shows the potential to improve the diagnosis and prognosis of breast cancer (47, 48). Genomewide tumor DNA methylation was measured using the Human Methylation 450K (HM450K) BeadChip array and Suman et al. (49) proved that ISM1 might serve as a biomarker for the prognosis of patients with lobular breast cancer that the methylation clusters were mainly located in the promoter region of the gene. Somatic activation mutations of the CTNNB1 gene are detected in adrenocortical adenomas and adenoid cystic carcinoma patients but without effective treatment (50–52). Researchers have found that the expression level of ISM1 was significantly upregulated in both CTNNB1 mutated adrenal tumors and CTNNB1 mutated adrenal lesions, suggesting that ISM1 may be a potential therapeutic target for CTNNB1 mutation-related adrenal disease (53). Briefly, ISM1 could be a potential therapeutic target for cancer.

### Cell growth

ISM1 can accelerate apoptosis mediated by both of two receptors with different mechanisms and there is no interaction between GRP78 and avb5 integrin. Xiang et.al found that ISM1 significantly inhibited VEGF-induced EC proliferation in a dose-dependent way *via* binding to αvβ5 integrin, but mildly inhibited serum-stimulated fibroblasts proliferation and had no effect on serum-stimulated tumor cell proliferation (19). Furthermore, ISM1 induced EC apoptosis mediated by αvβ5 integrin in the presence of VEGF, βFGF or serum and marginally induced fibroblast apoptosis in the presence of serum, but did not induce apoptosis of tumor cells (19). It seems that ISM1-αvβ integrin have a preferential effect on ECs proliferation and apoptosis. It is now well established that GRP78 exists on the cell surface of cancer cells and ECs and Chen et al. (38) found that ISM1 was a novel proapoptotic ligand that targeted cell-surface GRP78 to trigger apoptosis by inducing mitochondrial dysfunction (54, 55). Upon binding to GRP78, ISM1 is internalized into ECs through clathrin-dependent endocytosis that is essential for its proapoptotic activity. ISM1 co-targets with GRP78 to mitochondria where it interacts with ADP/ATP carriers on the inner membrane and blocks ATP transport from mitochondria to cytosol, thereby causing apoptosis. Indeed, ISM1 only induces significant apoptosis in cells that express high-level cell-surface GRP78 including HUVECs, HEK293T, B16F10, 4T1, 786-O and LS-LM6 cell lines (38).

### Permeability of endothelial cells

Emerging studies have shown that ISM1 was associated with permeability of ECs particularly in the lung. ISM1 protects lung homeostasis *via* cell-surface GRP78-mediated alveolar macrophage apoptosis, specifically, rISM1 effectively quenches lung inflammation while ISM1^−/−^mice develop spontaneous emphysema (56). Li et al. (57) uncovered that AECII-derived ISM1 participated in hypoxia-induced hyperpermeability of PMVEC monolayers, which was attenuated by small interference RNA of ISM1. Mechanistically, ISM1 is regulated by hypoxia-inducible factor-1a (HIF1a) which directly binding to the conserved regulatory elements upstream of the ISM1 locus. Accordingly, Venugopal et al. (58) reported that ISM1 induced EC monolayer permeability in a dose- and time-dependent manner through both cell-surface receptors, especially, ISM1 directly induced endothelial permeability through its receptor GRP78-mediated Src activation.

### Physiogenesis

ISM1 has also been shown to be required for asymmetric organ morphogenesis during development (18). Berrun et al. (59) demonstrated that ISM1 was required for normal generation of hematopoietic stem and progenitor cells and their downstream progeny during zebrafish hematopoiesis. Furthermore, some investigators determined that ISM1 was a more effective culture medium in generating higher quality embryos which reflected in the higher rate of clinical pregnancies and implantation and higher birth weight and height during the *vitro* culture period (60, 61). In addition, Li et al. (62) demonstrated that ISM1 was an age-related biomarker that decreased with senescence and could reverse the aging of *Nothobranchius guentheri*. However, the underling mechanism and its application in physiogenesis still need further research.

## Conclusion and perspective

In conclusion, we suggest ISM1 is distributed in different body compartments and plays critical roles in multiple biological processes as a newly defined adipokine. Nevertheless, the physiological function of ISM1 remains to be fully elucidated and there are still some challenges to be solved in future research. Firstly, future studies will be required to further address the underlying mechanism of its interaction with GRP78 and integrin, the downstream effector molecules of ISM1 receptors also need further study. It is critically important for ISM1 to clarify whether it behaves similarly when binding to different receptors, or whether other potential receptors exist. Secondly, we need to reveal the mechanism of ISM1shear release process and determine whether its roles are due to the pre-shear full length or post-shear mature ISM1. On the other hand, we should construction cell-specific transgenic mice and explore its autonomous roles. Great attention should be paid to the clinical significance and its indispensable roles in other tissues, such as in the cardiovascular system, immune system and musculoskeletal system. Finally, ISM1, a secretory factor, whether it can be detected in blood or other body-fluid, or serve as a biological marker for various diseases, remains elusive.

## Author contributions

MH, XZ, and Q-ZT contributed to the conception and design of the review. The draft of the manuscript was written by MH. XZ performed the literature search. CH, TT, and Q-ZT critically revised the manuscript. All authors contributed to the article and approved the submitted version.

## Funding

This work was supported by grants from National Natural Science Foundation of China (No. 81700254), the Key Project of the National Natural Science Foundation (No. 81530012), National Key R&D Program of China (2018YFC1311300), the Fundamental Research Funds for the Central Universities (No. 2042018kf1032), Development Center for Medical Science and Technology National Health and Family Planning Commission of the People's Republic of China (The prevention and control project of cardiovascular disease, 2016ZX-008-01), and Science and Technology Planning Projects of Wuhan (2018061005132295).

## Conflict of interest

The authors declare that the research was conducted in the absence of any commercial or financial relationships that could be construed as a potential conflict of interest.

## Publisher's note

All claims expressed in this article are solely those of the authors and do not necessarily represent those of their affiliated organizations, or those of the publisher, the editors and the reviewers. Any product that may be evaluated in this article, or claim that may be made by its manufacturer, is not guaranteed or endorsed by the publisher.
